# The Hospitals, &c., of New York

**Published:** 1859-12

**Authors:** 


### EDITORIAL DEPARTMENT.

### *The Hospitals, etc. of New York.*

Blackwell's Island.—The principal punitive and charitable institutions maintained by the inhabitants of New York city, and required for the detention and support of the numerous individuals in her immense population whose crimes or poverty render them a public charge, present an interesting subject of investigation; and it is the design of this sketch to give a short account of them. The location they occupy is admirably adapted for the purpose, sufficiently large for the present and prospective wants of the community; isolated from the city proper, so as to obviate many evils which accompany the erection of such buildings in a crowded neighborhood; and yet, from the facilities of transit, possessing all the advantages of a free and constant communication with the business center; and also offering the unfortunate inmates unequaled advantages of space and ventilation.

Blackwell's Island is situated in the East River, or Long Island Sound, within the corporate limits of New York, forming a part of the nineteenth ward of that city. Its superficial area is about one hundred and fifty acres; its length, from north to south, about one mile and three quarters, extending from a point opposite Forty-sixth street to Eighty-sixth street, and divided from the city by a channel some two hundred and eighty yards wide, which is also about the width of the channel separating it from Long Island. Up to the year 1817 or 1818, this island was in the possession of private parties, from whom the city purchased it, at a cost of thirty-five thousand dollars. It is a solid mass of gneiss mica, coarse granite, rising but a few feet above high-water mark, covered, in places, with a shallow stratum of soil. At the time of its purchase it was almost barren, some few rugged clumps of cedars, and equally rugged brush, being all that appeared to break the monotony of its stony surface. Upon it was erected a frame building, still existing, called "Blackwell House," situated about the center of the island, in the part now appropriated to the Alms-House Department. On its northeast point was a public gallows, where criminals were executed, among whom were Gibbs and Walnsley, the pirates.

It would be difficult to imagine a spot better adapted than Blackwell's Island to receive the *debris* of a large population. Situated in the midst of a deep and rapid river, and but a few miles from the open sea, it presents every facility for sanitary arrangements; with no excuse for insufficient drainage, and scarcely a possibility of any offensive accumulation of refuse matter. Its atmosphere is singularly pure and healthful. During the years 1856 and 1857, a meteorological observatory, in connection with the Smithsonian Institute, Washington, D. C., was in operation in the Hospital building; and from its published reports the following facts are taken:—

| | 1856. | 1857. |
|---|---|---|
| Mean Barometrical reading, . . . | 29.906 | 29.973 |
| Maximum " " . . . . | 30.617 | 30.943 |
| Minimum " " . . . . | 29.220 | 29.111 |
| Mean Thermometrical reading, . . | 49 | 50 |
| Maximum " " . . . | (July) 96 | (Aug.) 90 |
| Minimum " " . | (Jan'y) — 8 | (Jan'y) — 10 |
| Mean Temperature of Evaporation, . . | 44 | 46 |
| Relative Humidity (Saturation 1.00), . | .69 | .73 |
| Rain (Inches), . . . . . . | 40.230 | 55.824 |

Thus fitted by nature for the purpose to which it is applied, art and industry have not been wanting to complete the adaptability. Roads have been constructed upon its surface; made ground applied to the cultivation of vegetables required by its inmates; a substantial sea-wall constructed on much of its coast-line, and the rock, of which it consists, excavated in suitable places, to furnish material for the buildings required. Commencing at the southern extremity, the edifices may be enumerated as follows:—

1. Small-pox Hospital.

2. Island Hospital (in process of erection).

3. Penitentiary.

4. Female Alms-house.

5. Male Alms-house.

6. Work-house.

7. Island Hospital (temporary).

8. Lunatic Asylum.

In addition, there are a number of work-shops, cook-houses, wash-houses, stores, barns, and out-houses connected with the different buildings, two docks for landing heavy goods, and a ferry-house for the convenience of passengers.

The title to the land and buildings being vested in the city of New York, the management thereof devolves upon the corporation. These duties were performed, up to the year 1849, by the Common Council, with the assistance of a salaried Alms House Commissioner, chosen by the popular vote. The pay of this officer was three thousand dollars per annum; and the advocates of economy considered that a great victory was achieved when, on May 8, 1849, an unpaid board of ten citizens commenced the administration of the city's charities. In order to prevent the "Governors of the Alms House" becoming a party organization, it was devised that the members should be equally divided, so far as political views were concerned. The first Board was nominated by the State Legislature, and the act provided that two of the members should go out of office every year, and their successors be chosen by the people; and to maintain the balance of power, it was enacted that of the candidates for the vacancies, the one having the highest number of votes should be declared elected, and the one having the next highest number of votes should be appointed by the Mayor.

These pages are not the appropriate place to discuss either the politics or the policy of the Governors of the Alms House; and it will be more consonant with the reader's feelings to give a short sketch of the different institutions, and thus exhibit Blackwell's Island as it is.

Small-Pox Hospital.—Until the year 1856, patients afflicted with small-pox were treated in a collection of wooden shanties, standing a short distance northeast of the present commodious hospital. They presented no facilities for medical visitation and treatment; and the palpable inconvenience of the *only* small-pox hospital in New York city, forced the commencement of a building better suited for the purpose. The present hospital was opened for the reception of patients in January, 1857. In a speech delivered by Governor Isaac Townsend, on the occasion of its inauguration, he remarked:

"The small-pox hospital is of a smoothly cut, hard and durable blue stone, quarried on the island. The style is early English Gothic. It is 104 feet in length by 45 in breadth, three stories in height, resting on a heavy base, and surmounted by a bold stone cornice. The principal front, looking toward the city, is broken by a massive stone porch, surmounted by an oriel window. The north and south fronts have a stone bay window bracketed upon the second floor. The roof is of slate, pierced with clusters of tall chimney-shafts, and surmounted by an octagonal stone cupola.

"As to the interior arrangements: Two corridors cross each other on each floor, the entire length and width of the building. The staircase is of iron. The first floor is devoted to the use of the physician, matron, servants' rooms, kitchen, laundry and stores. The second floor is for charity patients, and is divided into four large and four small wards. The ceilings throughout are sufficiently lofty. Ventilation is secured by an air-duct connected with a chimney and wind-sail, and communicating, by flues in the wall, with each ward. Other flues connecting with the cupola are traversed by air that has been rarified by a constant fire and drawn from the rooms below." If to this be added that the third floor is divided into small rooms for private patients, that each room throughout the building is furnished with hot and cold water, marble mantels, and open fire-places, and each floor provided amply with baths and water-closets, enough is said to convey an idea of the small-pox hospital on Blackwell's Island, and to justify the remark of Governor Townsend: "We may venture to assume that we have erected, for the purposes of a small-pox hospital, a building which, for neatness, eligibility, and adaptation to its intended uses, will bear comparison with any similar establishment in the new or in the old world.

Before alluding to the professional results of this hospital, it is necessary to describe the general medical arrangements of Blackwell's Island. All the institutions enumerated, excepting the Lunatic Asylum, are under the medical charge of a physician, appointed by the Board of Governors, and designated, "Resident Physician of Blackwell's Island," whose authority extends to all questions relating to the health of the inmates. The present Resident Physician of the island, is Dr. William W. Sanger, who was appointed in 1853. The staff under his direction consists of a deputy Resident Physician and seven Assistant Physicians, selected from graduates of New-York colleges. These appointments are made by Dr. Sanger; each gentleman serves one year, his sphere of duties being so arranged as to give him a practical acquaintance with every department. Considering the large extent of the field, and the valuable opportunities it offers for acquiring a thorough knowledge of disease, it is not surprising that the list of applicants is always full, or that Dr. Sanger has an opportunity of selecting from the best informed and most talented young men of the day.

To resume.—The medical history of the Small-Pox Hospital is comprised in a small compass. The cases treated, and the mortality, are as follows:

| Years. | Treated. | Died. | Years. | Treated. | Died. |
|---|---|---|---|---|---|
| 1849 | 283 | 29 | 1855 | 61 | 10 |
| 1850 | 221 | 41 | 1856 | 137 | 27 |
| 1851 | 344 | 58 | 1857 | 208 | 33 |
| 1853 | 168 | 39 | 1858 | 243 | 40 |
| 1854 | 210 | 40 | | | |

The diseases causing death were as follows:

| Years. | Small-Pox. Confluent. | Small-Pox. Distinct. | Other Diseases. | Total. |
|---|---|---|---|---|
| 1849 | 28 | | 1 | 29 |
| 1850 | No particulars in report. | | | |
| 1851 | 44 | | 14 | 58 |
| 1853 | 35 | 4 | | 39 |
| 1854 | 23 | 8 | 9 | 40 |
| 1855 | 6 | 1 | 3 | 10 |
| 1856 | 21 | 5 | 1 | 27 |
| 1857 | 25 | | 8 | 33 |
| 1858 | 28 | 5 | 7 | 40 |

The ratio of mortality from Small-Pox was:

| | | | |
|---|---|---|---|
| 1848........ | 16.4 per cent. | 1854........ | 14.7 per cent. |
| 1849........ | 10.3 " | 1855........ | 18.9 " |
| 1850........ | 19.0 " | 1856........ | 18.9 " |
| 1851........ | 16.9 " | 1857........ | 12.0 " |
| 1853........ | 23.2 " | 1858........ | 13.5 " |

These tables of mortality present some facts worthy notice. Without being invidious, it may be stated that to the end of 1853, the mean annual mortality was 17.2 per cent. From the commencement of 1854, when the present Resident Physician assumed the charge, to the end of 1858, the average was 15.6 per cent.; and from January, 1857, when the new hospital was opened, to December, 1858, the mortality has averaged only 12.8 per cent., 4.8 per cent. less than in the old huts. This is enough to teach public officers that it is real economy to erect suitable buildings for the treatment of disease, supposing human life to possess any tangible value.

Allusion has been made already to the climate of Blackwell's Island; and some further extracts from Governor Townsend's Inaugural Address, will form a fitting conclusion to this notice of the Small-Pox Hospital:

"The climate of Blackwell's Island, and of the land interposed between the Sound and the ocean, depends more upon its insular character than upon its latitude. The influence of the sea renders it more temperate, more suitable for a hospital, than many other places in the very heart of our great continent, that are in the same range of latitude. Nor must we omit another consideration of great and happy value, that we have been enabled to isolate this hospital for contagious diseases, upon an island in the Sound which might seem to have been placed there *providentially* for this very purpose.

"Variableness of temperature, sudden atmospheric changes, constitute in a greater or less degree, a peculiarity of American climate. But these great alternations are modified somewhat in the neighborhood of New York, by the interposition of Long Island, over the surface of which (in summer) a breeze of cool and moist air from the ocean, fans (chiefly in the afternoon) the dry and heated ground, and renders it a comparatively agreeable and healthful residence during the hot months, while the same agency, in winter, melts the snow, often before it reaches the earth. Westerly and southwesterly currents of air prevail on the surface more than half the year, the thermometer seldom falling below zero, or rising above ninety, the mean temperature being about fifty-two degrees."

Island (late Penitentiary) Hospital.—Leaving the Small-Pox Hospital, and walking northward, we next encounter a large, but yet unfinished, building, destined for the Island Hospital. Until the close of 1857 this institution was known as the Penitentiary Hospital, and was, in fact, part of the Penitentiary. It is necessary the reader should remember this fact in perusing its history.

When the Penitentiary was erected it was designed to receive not only prisoners sentenced from the Courts of Sessions for positive crimes, but also vagrants arrested by the police, and committed by police justices. Among this latter class were included many drunkards and debased females, numbers of whom were suffering from syphilis or similar diseases, for whose treatment it was necessary to make provision. It is, however, reasonable to conclude that this necessity was not considered sufficient to justify the erection of hospital buildings at that time; and hence arose the fact that the Penitentiary Hospital was without a "local habitation," fitted for the reception and medical care of patients, until the commencement of the year 1850, although the systematic relief of suffering vagrants dates from the erection of the Penitentiary, in 1823. The germ of the present large hospital appeared in a small two-story shanty, upon the western edge of the island, a little to the south of the buildings now erecting. At the time, it was deemed that this accommodation would be ample for many years; but the hut soon became too small for the numbers who demanded its shelter, and a corresponding one was attached to it at right angles. This enlarged erection became, in turn, inadequate, so as to require another addition; which was provided, and the Penitentiary Hospital assumed the shape of three sides of a quadrangle.

Paradoxical as it may seem, the restrictions which had been devised to keep the sick from Blackwell's Island, by prohibiting medical treatment to any but inmates of the Penitentiary, had a very large share in compelling these additions to the hospital building. An unfortunate woman, arrested and committed for vagrancy, was found suffering from syphilis, transferred to the hospital, treated and cured before her sentence expired. Her arrest would be a fortunate occurrence, duly appreciated among those of her class who were suffering as she had suffered, and had no means of procuring professional assistance. Denied the privileges of the City Hospital, unless they could pay their board in advance, and absolutely refused admission to Bellevue, they had no resort but the Dispensary, and no home in which they could receive the visits of its physicians, or follow the treatment prescribed. The facile operation of intoxication, the subsequent arrest and committal, and the final reception into a hospital, were precisely adapted to their wants and wishes, and great numbers availed themselves of the facilities. Others deliberately walked into a police court, confessed themselves homeless vagrants, and asked to be committed to the Penitentiary for a shelter. In either case the result was the same, and hence arose one of the worst blots on the fair fame of New York; the fact that a homeless, friendless, moneyless man or woman, suffering from a loathsome and infectious disease, could obtain no relief except by becoming an inmate of the Penitentiary. This stigma is, at last, removed, and has left behind it, as a public benefit, the organization of a complete and well-regulated hospital, of easy access.

The wooden buildings mentioned were approrpiated for females, male patients being treated in a large room in the Penitentiary. So matters remained until 1848, when arrangements were made by the common council for the erection of a hospital; which was completed and occupied in 1850, and destroyed by fire in February, 1858. As an edifice, it reflected no credit either on its architect or builder; for hospital purposes, it had very few available recommendations. The plan embraced two wings, each 125 feet by 40 feet, and three stories high, and a middle house 50 feet square. The second and third floors of the west wing, and the second floor of the east wing, were each divided into five wards; the third floor of the east wing formed but one ward, and was the only really good hospital room in the building. The first, or basement stories were intended for wash-rooms and kitchens, but as patients increased, were gradually transformed into wards, despite their low ceilings and other disadvantages. There was no system of ventilation, except by the doors and windows, which were compelled to be closed in bad weather. The sewers connecting with the water-closets were so arranged, that every tide completely closed the outlets, and forced the effluvia into the wards; the closets themselves opening directly into the rooms at the east and west ends. In point of construction, little need be said. Within a year after its occupation, the walls were so insecure, that strong iron braces were inserted from side to side, and firmly secured by screws to prevent them falling. Either the foundation was insecure, or the workmanship most defective, or probably from both causes combined, the inhabitants always experienced a dread of some calamity. More than one grand jury of the city and county presented the building as unsafe; many architects surveyed it, and suggested appliances of strength; and several different plans were adopted from time to time. Its external appearance was massive; its fragile character but too palpably shown when the conflagration took place, and in less than three hours reduced it to a shapeless heap of ruins. As all the inmates were rescued from the flames, and the only loss was a large amount of property, both public and private, the destruction of the building must be considered a fortunate termination in comparison with the dreadful sacrifice which must have ensued had it fallen.

Upon the site occupied by this structure, the Governors of the Alms

House are now erecting an edifice, which they promise shall be as complete as science and experience can devise. Accommodations are to be provided for about nine hundred patients; every improvement with regard to light, heat, ventilation, and drainage will be made available, and the hospital, when complete, is to be the model hospital of the United States.

Until 1846, the Penitentiary Hospital (as also the Small-pox Hospital) was under the management of the resident physician of Bellevue Hospital, who was accustomed to detail one or more members of his staff to attend it. In its earlier stages, the physician charged with this duty left Bellevue every morning, went to his labor in the Alms-House boat, and after making his rounds returned to Bellevue in time for dinner; a striking contrast to the medical arrangements at the present day. In 1846 the common council severed this connection, and appointed Dr. W. W. Sanger (the present incumbent), resident physician of Blackwell's Island. Dr. Sanger held the office until 1848, when he resigned and was succeeded by Dr. William Kelly; who in turn resigned in October, 1853, and was succeeded by Dr. Sanger, who returned to the scene of his earlier labors to find the circle very much enlarged, and demanding the exercise of all his administrative talent.

One of Dr. Sanger's first attempts was, to provide an efficient system of ventilation. In the upper story of each wing he erected an air tight chamber, in which a furnace was placed. From this chamber ran a series of tubes, about nine inches square, communicating with every department of the building, and by the rarefaction of the atmosphere caused by the furnace, effectually drawing all the foul air into the air-tight chamber, whence it escaped through a lofty cupola provided for the purpose. This system of exhaustion answered admirably. The position of the hospital rendered unnecessary any plan for the introduction of fresh air, as enough always found entrance through the doors and windows. Another improvement was the reconstruction of the sewers, and an alteration of the water-closets, removing the latter some feet from the main building, and providing for access an ante-room, of which the windows were kept open except in very stormy weather. The wash-rooms and kitchens were removed to a detached building, which also contained a large steam boiler for heating water in the hospital tanks, and generating steam for washing and cooking, and a steam engine pumping water from the river into the main building, thus affording opportunities for both salt and fresh water baths, either hot or cold. In short, Dr. Sanger did everything in his power to transform an unsuitable into a suitable hospital.

The number of patients treated, and the amount of mortality at the

Penitentiary, or Island, Hospital during the last ten years is stated as follows, in the published reports of the Board of Governors:

| *Years.* | *Treated.* | *Died.* | *Years.* | *Treated.* | *Died.* |
|---|---|---|---|---|---|
| 1849 | 2,403 | 292 | 1854 | 4,058 | 144 |
| 1850 | 2,201 | 80 | 1855 | 2,657 | 66 |
| 1851 | 2,541 | 97 | 1856 | 2,083 | 38 |
| 1852 | 3,034 | 111 | 1857 | 3,157 | 74 |
| 1853 | 3,136 | 109 | 1858 | 4,676 | 113 |

The deaths in 1849 include 122 from cholera. The ratio of mortality, calculated on the gross number of patents, is as follows:

| | | | |
|---|---|---|---|
| 1849 | $12\frac{1}{10}$ pr. ct. | 1854 | $3\frac{5}{10}$ pr. ct. |
| 1850 | $3\frac{1}{10}$ | 1855 | $2\frac{5}{10}$ |
| 1851 | $3\frac{8}{10}$ | 1856 | $1\frac{8}{10}$ |
| 1852 | $3\frac{6}{10}$ | 1857 | $2\frac{3}{10}$ |
| 1853 | $3\frac{5}{10}$ | 1858 | $2\frac{4}{10}$ |

During the year 1849, cholera and ship fever were epidemic. The deaths from these diseases alone were 141, which will reduce the ratio from $12\frac{1}{10}$ per cent. to $6\frac{6}{10}$ per cent. Also in 1854 cholera was epidemic, and 14 deaths resulted from it, reducing the ratio of mortality from $3\frac{5}{10}$ per cent. to $3\frac{2}{10}$ per cent. With these corrections, the following results are obtained:

Average mortality from 1849 to 1853........$4\frac{1}{10}$ per cent.

" " " 1854 " 1858........$2\frac{4}{10}$ "

Or a decrease of $1\frac{7}{10}$ per cent. per annum, in the ratio of mortality since the improvements of the hospital building were commenced.

Every medical man will admit that, for professional purposes, it is not sufficiently accurate to calculate the ratio of mortality from the total number of deaths occurring in a public hospital. There are always a greater or less number of cases resulting from causes which no physician can influence,—cases which only reach his hands when all hopes of palliation are vain. In common justice to the profession these should not be allowed to swell the list; and, accordingly, the Resident Physician of Blackwell's Island deducts them, and presents what he considers the mortality resulting from the practice of the hospital under his charge, in the following form:

1854........$3\frac{3}{10}$ per cent. (including cholera).

1855........$2\frac{3}{10}$ "

1856........$1\frac{6}{10}$ "

1857........$2\frac{1}{10}$ "

1858........$2\frac{1}{10}$ "

It is matter of regret that this minuteness of detail is not generally observed in reports of public medical institutions. It tends to give the profession at large a far better idea of the results of their practice.

The annexed table gives the causes of death in 1858:

| | |
|---|---|
| Abcess | 1 |
| Albuminuria | 1 |
| Apoplexy | 2 |
| Arachnitis | 1 |
| Ascites | 2 |
| Brain, Inflammation of | 1 |
| Cholera Infantum | 1 |
| Convulsions | 1 |
| Coroner's Cases | 6 |
| Croup | 1 |
| Debility | 2 |
| Delirium Tremens | 10 |
| Diarrhœa | 4 |
| Dysentery | 1 |
| Epilepsy | 1 |
| Erysipelas | 2 |
| Fever, Puerperal | 2 |
| " Typhoid | 2 |
| Heart, Fatty degeneration of | 1 |
| " Hypertrophy of | 1 |
| " Valvular Disease of | 4 |
| Hemiplegia | 1 |
| Kidneys, Fatty degeneration of | 2 |
| Liver, Cirrhosis of | 2 |
| Marasmus | 2 |
| Meningitis | 6 |
| Phthisis Pulmonalis | 41 |
| Pneumonia | 10 |
| Syphilis, Tertiary | 2 |
| | 113 |

The Blackwell's Island Hospital offers, unquestionably, the best field in the United States for the study of diseases of the generative organs, and is, excepting the Parisian Hospitals, probably the largest in the world. From 1854 to 1858, the venereal and uterine cases treated were as follows:

| VENEREAL. | 1854. | 1855. | 1856. | 1857. | 1858. |
|---|---|---|---|---|---|
| Balanitis | 11 | 12 | 11 | 32 | 58 |
| Bubo | 162 | 161 | 201 | 223 | 338 |
| Condylomata | 31 | 56 | 51 | 44 | 73 |
| Gonorrhœa | 90 | 154 | 209 | 162 | 221 |
| Orchitis | 12 | 18 | 14 | 27 | 31 |
| Phagedæna | 33 | 32 | 21 | 44 | 58 |
| Rupia | " | 9 | 22 | 33 | 41 |
| Stricture of Urethra | 4 | 7 | 9 | 25 | 28 |
| Syphilis, Primary | 606 | 660 | 652 | 882 | 1,206 |
| " Secondary | 287 | 196 | 208 | 352 | 453 |
| " Tertiary | 119 | 92 | 29 | 74 | 128 |
| Syphilitic Vegetation | 44 | 56 | 68 | 83 | 140 |
| UTERINE. | | | | | |
| Amenorrhœa | 52 | 112 | 39 | 41 | 74 |
| Cervix Uteri, Hypertrophy of. | 106 | 175 | 43 | 39 | 53 |
| " " Inflammation of | 133 | 63 | 32 | 36 | 64 |
| " " Induration of | 72 | 113 | 29 | 61 | 57 |
| " " Ulceration of | 117 | 255 | 224 | 166 | 218 |
| Leucorrhœa | 14 | 5 | 10 | 12 | 36 |
| Menorrhagia | 27 | 14 | 10 | 6 | 15 |
| Ovaritis | 4 | 1 | 3 | .. | .. |
| Prolapsus Uteri | 16 | 17 | 2 | 19 | 22 |
| Vaginitis | 132 | 117 | 45 | 105 | 138 |

Although these form such a large amount, it must not be supposed they are the sole maladies treated. In the report for 1858, the following appear among other diseases:

| | | | |
|---|---|---|---|
| Abscess | 43 | Fractures | 18 |
| Adenitis | 19 | Heart, Fatty degeneration of | 1 |
| Albuminuria | 12 | " Hypertrophy of | 4 |
| Aneurism | 5 | " Valvular disease of | 14 |
| Apoplexy | 5 | Hernia | 34 |
| Ascites | 6 | Hemorrhoids | 83 |
| Asthma | 9 | Injuries | 138 |
| Bronchitis | 121 | Iritis | 58 |
| Carbuncle | 6 | Kidney, Fatty degeneration | 2 |
| Caries | 13 | Meningitis | 6 |
| Conjunctivitis | 34 | Necrosis | 9 |
| Debauch | 976 | Ophthalmia | 19 |
| Delirium Tremens | 305 | Paralysis | 24 |
| Diarrhea | 116 | Parturition | 19 |
| Dysentery | 30 | Pericarditis | 4 |
| Endocarditis | 1 | Phthisis Pulmonalis | 239 |
| Enteritis | 2 | Pleuritis | 82 |
| Erysipelas | 25 | Pneumonia | 46 |
| Epilepsy | 22 | " Pleuro | 1 |
| Fever, Intermittent | 85 | " Typhoid | 2 |
| " Puerperal | 2 | Rheumatism | 280 |
| " Remittent | 6 | Scrofula | 11 |
| " Typhoid | 3 | Synovitis | 7 |
| Fistula in Ano | 7 | Tonsillitis | 18 |
| " Recto-vaginal | 6 | Ulcers | 301 |

The census of the Hospital shows, as the average daily number of inmates.

| | | | |
|---|---|---|---|
| 1854 | 418. | 1857 | 396. |
| 1855 | 380. | 1858 | 392. |
| 1856 | 332. | Average for five years | 384. |

It only remains to say, before closing this notice of the Island Hospital, that its female patients are now treated in a building erected for a factory, in connection with the Work-house, near the north end of the Island, and the males in rooms in the Work-house building. This arrangement, of course, presents many inconveniences; but it is only temporary, as the new building will be completed in the course of the year 1860.

Penitentiary.—When this building was erected, in 1823, it was designed for the reception of two classes of inmates,—vagrants, already mentioned; and those sentenced from the courts of general or special sessions fo r petty larceny, misdemeanors, or assault and battery. This a rangement continued in force until the Work-house was completed; the only distinction made between the prisoners being in costume: those committed by police magistrates were dressed in a rough suit of brown, and those sentenced from court wore a striped uniform. Up to quite a recent period, the general management of the charitable and punitive institutions of New York, seems to have been adapted to degrade all who needed charity, and force them to become criminals; and it can scarcely be denied that the project was successful. Those who have investigated the subject will be fully prepared to account for the rapid increase of the minor crimes which disfigure the calendars of our criminal courts.

The Penitentiary is a long, dreary-looking structure of light-colored stone, relieved only by a centre building intended for a warden's residence, but now devoted to offices, keeper's rooms, etc. The walls rise about fifty feet, are castellated on the top, and pierced by four ranges of narrow loop holes guarded by iron bars. The interior arrangement is three double tiers of cells in the centre of the building. The doors of the cells face toward the outer walls; those on the lower tier open directly on the flagging of the prison, those on the upper tiers on iron galleries bracketed from the walls. Each cell is numbered and intended for but one prisoner; although when the building is crowded, two are frequently forced to occupy one apartment. The only furniture in the cell is an iron bed-frame upon which canvas is stretched, and one or more blankets according to the season. A theoretical attempt at ventilation has been unsuccessfully made; the heavy miasma will not ascend in the pipes provided for the purpose, because there is no auxiliary aid to exhaust it, and for at least twelve hours out of every twenty-four, the prisoners breathe an atmosphere vitiated by some hundreds of persons. With no attempt at drainage, and an abortive system of ventilation, it is unnecessary to describe the sensation experienced on entering this building on a hot summer morning. For some time the Penitentiary has been much overcrowded, and a new wing is now erecting, which is promised to be arranged in a manner that will overcome some of the nuisances of the old building.

It will be remembered that the hospital formed a part of the Penitentiary until the commencement of 1858; and, consequently, up to that time no separate record was kept of its medical business, patients who required assistance being transferred, and their cases appearing on the general hospital books. Now a room is appropriated for invalids, and the report for 1858 gives a list of the diseases treated therein. Lues Venerea has its full share, as the following figures show:

| | | | |
|---|---|---|---|
| Balanitis | 13 | Stricture of Urethra | 22 |
| Bubo | 13 | Syphilis Primary | 279 |
| Gonorrhœa | 103 | " Secondary | 292 |
| Orchitis | 32 | " Tertiary | 8 |
| Phagedæna | 1 | Syphilitic Vegetations | 18 |

Of general diseases there were, among others,

| | | | |
|---|---|---|---|
| Abscess | 41 | Fever, Intermittent | 65 |
| Bronchitis | 39 | Fractures | 17 |
| Cancer | 4 | Injuries | 176 |
| Cholera Morbus | 41 | Phthisis Pulmonalis | 74 |
| Diarrhœa | 104 | Pleurisy | 15 |
| Dysentery | 13 | Pneumonia | 25 |
| Erysipelas | 4 | Ulcers | 174 |

The ratio of mortality was four tenths of one per cent. on the cases treated, the number of deaths being 9, from the following causes :

| | | | |
|---|---|---|---|
| Apoplexy | 1 | Meningitis | 1 |
| Brain, Congestion of | 1 | Phthisis Pulmonalis | 3 |
| Dysentery | 1 | Pneumonia | 1 |
| Epilepsy | 1 | | |

Among the members of Dr. Sanger's staff, the Penitentiary is considered a valuable field of practice, not so much for actual skill in treatment, as for the necessity it enforces of a correct diagnosis, and the detection of numerous "malingerers," who crowd the doctor's clinique in hopes of obtaining respite from labor.

Alms Houses.—Still journeying northward, the next buildings which require attention are the Alms Houses, especially devoted for the pauper population of New York, and containing at the present time some sixteen hundred inmates. Here may be met the extremes of human life,—decrepid senility on the verge of the grave, and sickly infancy just flickering into existence; the really honest but destitute, and the incorrigibly idle and worthless. Walking through the grounds, the visitor sees much that excites his sympathy, and much to convince him that many of the recipients of public charity receive an alms for which they have no claim.

The medical business of the Alms Houses, where two of Dr. Sanger's assistants are stationed, includes a very large amount of infantile and chronic disease. The list of maladies treated is too long to reprint; but the following extract from the Report for 1858, will give the medical world an idea of the extent of this practice. Among the cases are found :—

| | | | |
|---|---|---|---|
| Abscess | 172 | Hernia | 61 |
| Adenitis | 44 | Hydrocephalus | 13 |
| Apthæ | 22 | Iritis | 111 |
| Ascites | 20 | Marasmus | 192 |
| Bronchitis—chronic | 834 | Ophthalmia | 232 |
| Cholera Infantum | 115 | Paralysis | 118 |
| Cholera Morbus | 231 | Phthisis Pulmonalis | 737 |
| Conjunctivitis | 441 | Pleurisy | 151 |
| Convulsions | 25 | Pneumonia | 294 |
| Croup | 10 | Purpura | 228 |
| Diarrhœa | 1785 | Rheumatism | 690 |
| Dysentery | 196 | Rubeola | 85 |
| Epilepsy | 42 | Scorbutus | 66 |
| Fever, Intermittent | 392 | Scrofula | 16 |
| Granular Lids | 25 | Stomatitis | 79 |
| Heart, Hypertrophy of | 17 | Synovitis | 88 |
| " Valvular disease of | 37 | Ulcers | 913 |

The total number of deaths in 1858 was 393, a ratio of four per cent. on the cases treated.

For several years the Resident Physician of Blackwell's Island has complained of the custom of the authorities at Bellevue Hospital, who make a habit of transferring to the Alms Houses a large number of incurable cases. In 1857, of 319 cases resulting in death, 49 were received from Bellevue Hospital direct, in addition, doubtless, to a large proportion who were discharged from the Hospital, and, after remaining a day or two in New York, supplicated admission to the Alms House to die. Of 393 patients who died in the Alms House in 1858, 41 were received direct from Bellevue Hospital. Dr. Sanger remarks upon this subject in his Report for 1857:

"The summary which precedes the mortality table will show you that 49, or 15 4-10 per cent. of those who died were received in the Alms House direct from Bellevue Hospital. A large portion of those coming from New York were, the day preceding their admission here, discharged from the City Hospitals proper, principally from Bellevue. The Report for 1854, in allusion to this matter, says:—'I can see no reason for making the Alms House a "Dead House" for other institutions, nor can I convince myself that it is merciful to transfer persons in, or near, a dying condition, from one institution to another.' Subsequent observations have not induced me to change this opinion. The system bears unduly upon the Alms House, making that department responsible for the death of persons who have been treated elsewhere."

Work House.—The New York Work House was established by an act of the State legislature of 1849; the same body that created the Board of Alms House Governors. The project was based upon the fact that many inmates of the Alms House were able, and the assumption that they were willing, to work; and the theory has been signally falsified by the subsequent progress of the institution. Many believed it would become eventually self-supporting. So far from this expectation being realized, it is maintained by the tax-payers at an expense of about fifty thousand dollars per annum.

The Work-House buildings were commenced in 1850, upon plans prepared by James Renwick, Jr., Esq., and were completed in 1855, at a cost of nearly two hundred thousand dollars. The interior arrangement consists of small cells, rising in tiers like those of the Penitentiary, with this difference: instead of two ranges being placed back to back down the center of the building, they are placed on each side so that the exterior walls form the back of the cells, and the doors open opposite to each other, into a broad corridor on the lower floor, and to iron galleries on the upper tier. This makes the cells far more comfortable and habitable, and removes many of the nuisances already described.

As soon as the Work House was ready for occupancy, the Board of Governors decided to transfer there the vagrants who had, hitherto, been mingled with criminals in the Penitentiary; and to this use the Work House has been appropriated ever since. The attempt to populate it with industrious paupers was found futile, and it now depends upon police magistrates to keep it supplied with inmates. Occasionally a man or woman wishing shelter within its walls, applies to the Superintendent of Out Door Poor for admission; but the chief part of its denizens are those arrested by police officers. At the present time, two thirds of the patients in the Island Hospital are those who have been committed to the Work House as vagrants.

The medical business of the Work House is but small, as cases requiring treatment are transferred to the hospital. There are two large rooms appropriated for those afflicted with chronic diseases, who are not legitimate subjects for hospital treatment. The daily Work-House clinique is interesting to the profession. Every person admitted is examined by the physician detailed for that duty, and he decides who shall be transferred to the Hospital. The annual number admitted ranges from four to five thousand, among whom are found many different forms of disease; and their examination cannot fail to be valuable and instructive.

Lunatic Asylum.—Passing the building temporarily occupied as the Island Hospital, the Lunatic Asylum is reached. This is situated near the northern extremity of the Island. In priority of erection it stands second, the central portion of it having been built soon after the Penitentiary. It is under the charge of Dr. M. Harris Ranney, who has been resident physician for many years, and has discharged his duties in a manner which, at once, reflects great credit upon his abilities, and affords the most perfect satisfaction to all who visit the institution. Dr. Ranney is assisted by a salaried deputy resident physician, and one assistant physician. In his practice he is an ardent disciple of the humane methods now, almost universally, adopted in asylums for the insane. Force he never resorts to, unless under circumstances where the patient would injure himself or others if left at liberty. The results of his labors are most encouraging.

Below will be found a statement of the number under treatment each year.

| | | | |
|---|---|---|---|
| 1849 | 996 | 1855 | 926 |
| 1850 | 792 | 1856 | 939 |
| 1851 | 905 | 1857 | 923 |
| 1853 | 1014 | 1858 | 982 |
| 1854 | 1028 | | |

The following table gives a more comprehensive view of the business of the Lunatic Asylum, as the mere number under treatment affords no data upon which to base opinions.

| *Years.* | *Admitted.* | *Discharged.* | *Died.* | *Years.* | *Admitted.* | *Discharged.* | *Died.* |
|---|---|---|---|---|---|---|---|
| 1849 | 459 | 283 | 212 | 1855 | 371 | 253 | 100 |
| 1850 | 391 | 251 | 77 | 1856 | 366 | 276 | 66 |
| 1851 | 441 | 308 | 80 | 1857 | 326 | 221 | 75 |
| 1853 | 487 | 357 | 115 | 1858 | 355 | 235 | 92 |
| 1854 | 486 | 283 | 190 | | | | |

The results of Dr. Ranney's treatment are as follows:

| *Years.* | *Recovered.* | *Improved.* | *Unimproved.* | *Total discharged.* |
|---|---|---|---|---|
| 1849 | 212 | 60 | 11 | 283 |
| 1850 | 179 | 64 | 8 | 251 |
| 1851 | 208 | 90 | 10 | 308 |
| 1853 | 271 | 71 | 15 | 357 |
| 1854 | 186 | 65 | 32 | 283 |
| 1855 | 200 | 42 | 11 | 253 |
| 1856 | 174 | 81 | 21 | 276 |
| 1857 | 152 | 51 | 18 | 221 |
| 1858 | 164 | 54 | 17 | 235 |

The average number of patients in the Lunatic Asylum is 700. This is more than the accommodations will warrant, and it is a satisfaction to know that measures are in progress for the erection of an additional wing. As every true philanthropist must rejoice at the care and kindness now bestowed upon the unfortunate bereft of reason, so must all admit that nothing can add more to their comfort, or advance the labors of science in their behalf, than properly arranged and roomy buildings. Insanity cannot be aggregated with any expectation of a beneficial result; for it is then an approximation to the old system, which considered merely a safe detention.

It would not be courtesy to the officers, both medical and non-medical, of Blackwell's Island, to conclude this sketch without an acknowledgment of the politeness they show to visitors of the various departments.

